# Viral Infections, the Microbiome, and Probiotics

**DOI:** 10.3389/fcimb.2020.596166

**Published:** 2021-02-12

**Authors:** Ashton Harper, Vineetha Vijayakumar, Arthur C. Ouwehand, Jessica ter Haar, David Obis, Jordi Espadaler, Sylvie Binda, Shrilakshmi Desiraju, Richard Day

**Affiliations:** ^1^ ADM Health & Wellness, Medical Affairs Department, Somerset, United Kingdom; ^2^ Global Health and Nutrition Sciences, DuPont Nutrition and Biosciences, Kantvik, Finland; ^3^ Scientific Committee, IPA, Los Angeles, CA, United States; ^4^ Innovation Science & Nutrition Department, Danone Nutricia Research, Palaiseau, France; ^5^ Innovation, AB-BIOTICS S.A., Barcelona, Spain; ^6^ Lallemand Health Solutions, Montreal, QC, Canada; ^7^ Triphase Pharmaceuticals, Pvt Ltd, Karnataka, India

**Keywords:** microbiome, probiotics, viral infection, immunity, dysbiosis

## Abstract

Viral infections continue to cause considerable morbidity and mortality around the world. Recent rises in these infections are likely due to complex and multifactorial external drivers, including climate change, the increased mobility of people and goods and rapid demographic change to name but a few. In parallel with these external factors, we are gaining a better understanding of the internal factors associated with viral immunity. Increasingly the gastrointestinal (GI) microbiome has been shown to be a significant player in the host immune system, acting as a key regulator of immunity and host defense mechanisms. An increasing body of evidence indicates that disruption of the homeostasis between the GI microbiome and the host immune system can adversely impact viral immunity. This review aims to shed light on our understanding of how host-microbiota interactions shape the immune system, including early life factors, antibiotic exposure, immunosenescence, diet and inflammatory diseases. We also discuss the evidence base for how host commensal organisms and microbiome therapeutics can impact the prevention and/or treatment of viral infections, such as viral gastroenteritis, viral hepatitis, human immunodeficiency virus (HIV), human papilloma virus (HPV), viral upper respiratory tract infections (URTI), influenza and SARS CoV-2. The interplay between the gastrointestinal microbiome, invasive viruses and host physiology is complex and yet to be fully characterized, but increasingly the evidence shows that the microbiome can have an impact on viral disease outcomes. While the current evidence base is informative, further well designed human clinical trials will be needed to fully understand the array of immunological mechanisms underlying this intricate relationship.

## Introduction

Viruses (from the Latin “virus,” poison) are the most ubiquitous and abundant of all evolutionary entities (Norrby, 2008). We may never know how many unique viruses exist on Earth, however ~320,000 types of viruses are estimated to infect mammals (~5,500 species) alone (Anthony, 2013), and >1,000 unique viruses are known to infect humans (Lasso et al., 2019). Although the origin of viruses is contentious, it is fascinating to consider that they may have predated the Last Universal Cellular Ancestor (~4 billion years old) and played a substantial role in the evolution of life on Earth; in particular our own (at least 8% of the human genome is composed of sequences related to infectious retroviruses) (Moelling and Broecker, 2019).

Viral infections attack a wide range of tissues and organs including: the upper respiratory tract and lungs (e.g., rhinoviruses and influenza), the colon (e.g., rotavirus), the liver (e.g., Hepatitis B virus), the spinal cord (e.g., Poliovirus), vascular endothelial cells (e.g., Ebola), and white blood cells (e.g., HIV). Viral infections have been responsible for an astounding number of deaths in human history. Smallpox (caused by variola virus), which was successfully eradicated in 1980, is estimated to have caused 300–500 million deaths in the 20th century alone (Thèves et al., 2014). HIV has infected 75 million people and killed ~32 million of them to date (https://www.who.int/gho/hiv/en/). And, as of December 1^st^ 2020, severe acute respiratory syndrome coronavirus 2 (SARS CoV-2) has infected >63 million, killed >1.4 million while cases are increasing fast. The effects of the disease also cause profound damage to the world economy.

In common with all metazoans (multicellular, mitochondrial eukaryotes) we are holobionts; hosts with associated communities of microorganisms (Simon et al., 2019). The assemblage of microorganisms (bacteria, archaea, lower and higher eukaryotes, and viruses) present in a defined environment defines the term microbiota. Microbiome refers to the entire habitat and surrounding environmental conditions of a given microbiota and their collective genomes (Marchesi and Ravel, 2015). The human gastrointestinal (GI) tract alone contains 4 × 10^13^ bacteria, slightly more than the total number of our own cells (Sender et al., 2016). The term hologenome (holobiont genome) describes the combination of the host genome (humans ~20,000 genes) and associated collective microbial genomes (>33 million genes) (Simon et al., 2019); 9 million of which are unique protein-coding genes (Yang et al., 2009). Our microbial residents thus substantially expand our genetic repertoire and enable us to navigate our environment in ways that we would not be able to alone.

The Human Microbiome Project (HMP) launched in 2007 by the National Institutes of Health, enabled the characterization and understanding of the importance of the microbiome in human health and disease (Turnbaugh et al., 2007). Furthermore, eukaryotic double-stranded DNA (dsDNA) viruses have been analyzed by looking at the metagenomic sequence data generated by the HMP. The study detected on average 5.5 viral genera in each individual and reported high interpersonal diversity (Wylie et al., 2014). Studies on the human virome are relatively limited in number due to challenges such as; lack of common markers for viruses, the heterogeneity of the virome elements, low biomass samples, confounding by host DNA background, lack of standard computational tools for analysis of the virome, and an exponentially growing virome database. However, a limited number of virome studies suggest that viruses may have either beneficial or detrimental effects on human health, depending on their interactions with the host, other viruses, and bacteria. For example, it was observed that specific expansion of Caudovirales (bacteriophages) in Crohn’s disease has been associated with decreased bacterial diversity, supporting the idea that the virome may contribute to intestinal inflammation and bacterial dysbiosis (Norman et al., 2015). While many of the human virome studies report on correlation between virome fluctuations and certain diseases, the mechanistic understanding is limited and we do not know whether these altered viromes actually contribute to disease or merely correlate with it. On the other hand, some viruses may be protective; for example, emerging evidence indicates that the hepatitis G virus (HGV) could have a protective effect against human immunodeficiency virus (HIV)-associated disease (Lanteri et al., 2015). Human virome in health and disease is still in its infancy and knowledge of the precise mechanisms by which viruses could provide protection are limited. Although beyond the scope of this current review, a number of other recent review papers have explored the role of the human virome in health and disease indicating the growing interest in the human virome and its wider impact (Zou et al., 2016; Neil and Cadwell, 2018; Adiliaghdam and Jeffrey, 2020).

Leaving the human virome to one side and returning to the wider human GI microbiome, this has been repeatedly shown to be an important variable in human health, given its contribution to multiple aspects of human physiology including digestion, vitamin synthesis, and development and maintenance of the immune system (Olszak et al., 2012; Yatsunenko et al., 2012). The impact on human health and disease of microbiomes in other anatomical niches, such as the vagina (Smith and Ravel, 2017), lungs (Dickson and Huffnagle, 2015) and skin (Byrd et al., 2018), is also substantial.

A large variety of factors act to shape and potentially disrupt individual microbiomes, and these can be broadly divided into intrinsic factors (or nature), such as genetics, aging and physiological parameters (e.g., pH, oxygen levels, motility, hormonal fluctuations), and extrinsic factors (or nurture/exposure), such as diet, infections and medications (Levy et al., 2017). Given that certain microbiome states may increase susceptibility to infection and disease (Mcburney et al., 2019), interest has grown in the utility of probiotics (“live microorganisms that, when administered in adequate amounts, confer a health benefit on the host”) (Hill et al., 2014), which offer the potential to beneficially alter the microbiome to enhance antiviral immunity.

This review is divided in three sections exploring 1) the interplay between the microbiome, the host immune system and viral pathogens, 2) how various exposures and life events (e.g., diet, medications, aging etc.) disrupt the microbiome and impact on viral immunity, and 3) how the manipulation of the microbiome with probiotics may prevent or ameliorate a range of viral infections.

## The Microbiome and Probiotic Antiviral Mechanisms

**Figure 1:**
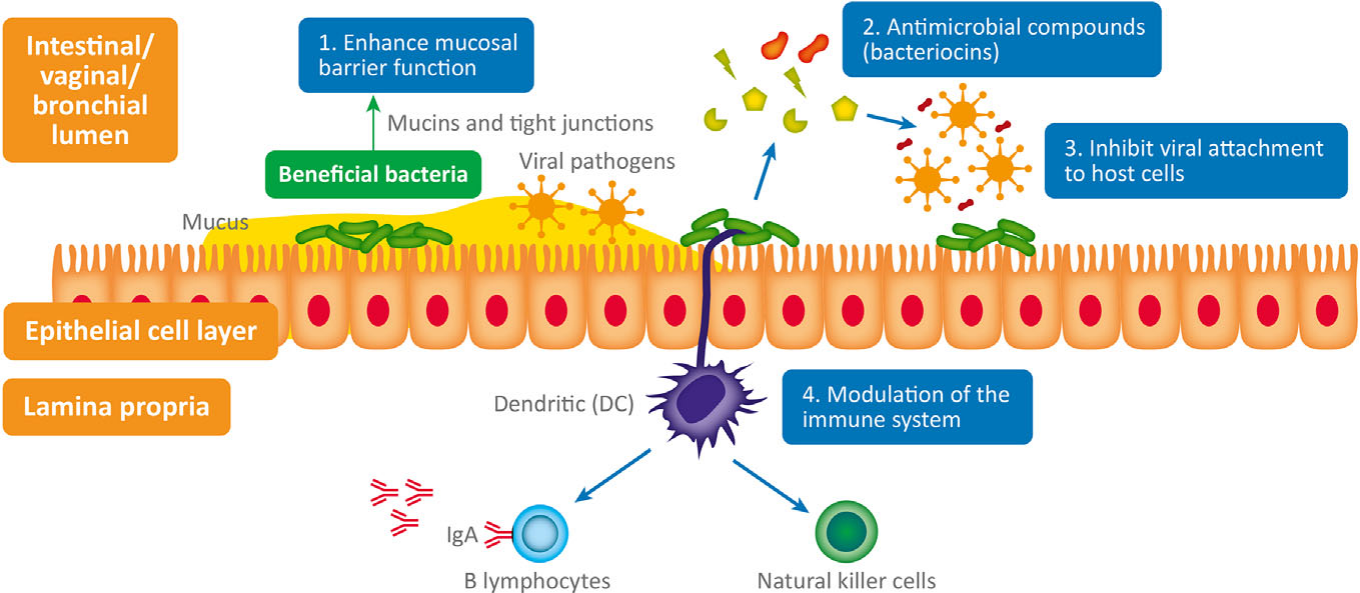
Antiviral microbiome mechanisms: 1. Enhanced mucosal barrier function: wet epithelial surfaces (e.g., GI tract, vagina, lung) are covered in a mucous layer containing glycoproteins - called mucins – which provide a physical barrier between invasive pathogenic microorganisms and host epithelial cells. Mucin production is influenced by the composition of the GI microbiome and they appear to have antiviral properties (Lieleg et al., 2012; Schroeder, 2019). Numerous lactic acid bacteria have been shown to regulate tight junctions and thus maintain normal mucosal permeability, 2. Antimicrobial compounds (bacteriocins) produced (e.g., subtilosin) by some bacteria (e.g., *Bacillus* spp.) are virucidal (Torres et al., 2013; Quintana et al., 2014). 3. Inhibit viral attachment to host epithelial cells by various means (Botić et al., 2007; Su et al., 2013). 4. Modulation of the immune system: probiotics stimulate plasmacytoid dendritic cells to produce interferon-alpha (IFN-a) thus inducing the cytotoxic activity of natural killer (NK) cells (innate immune system), which play a critical role in viral infection (Jounai et al., 2012). Probiotic bacterial strains have also been shown to enhance the production of antiviral immunoglobulins (produced by B lymphocytes – adaptive immune system) by an unknown mechanism; possibly through stimulation of intestinal epithelial cells or immunocytes (De Vrese et al., 2005).

The interplay between bacteria, viruses and host physiology is complex, and we still have much to learn. Despite this, a mounting body of evidence is beginning to reveal the fascinating contribution of both commensal and probiotic organisms to host defense against viral pathogens (Li N et al., 2019). When virus are exposed to mucosal surfaces (e.g., vaginal, respiratory, or GI) they have three broad lines of defense to overcome: the mucus layer, innate immune defenses and adaptive immune defenses (Kumamoto and Iwasaki, 2012). Evidence suggests that various commensal and probiotic bacteria influence each of these lines of defense with important relevance to a range of viral infections. The antiviral mechanisms responsible (summarized in **Figure 1**) are both direct and indirect and include: 1) Enhanced mucosal barrier function (Lieleg et al., 2012; Schroeder, 2019), 2) Secretion of antiviral antimicrobial peptides (AMPs) (Torres et al., 2013; Quintana et al., 2014); bacteriocins, 3) Inhibition of viral attachment to host cells (Botić et al., 2007; Su et al., 2013), 4) Modulation of antiviral innate and adaptive leucocyte function (De Vrese et al., 2005; Jounai et al., 2012).

The mucosal epithelium is a vital barrier against pathogenic viruses and bacteria. The first component of this barrier is mucus; a porous biopolymer matrix that coats all wet epithelial surfaces in the human body (e.g., vagina, GI tract, and lungs) (Lieleg et al., 2012). Although the mucus layer was once thought to be a passive, strictly host-controlled structure, recent research has demonstrated that the form and function of the mucus layer is influenced by the microbiome (Schroeder, 2019). In the GI tract, for instance, the microbiota induces expression of the genes encoding mucin 2; the main constituent of the glycoprotein network of GI mucus (Schroeder, 2019). Interestingly, porcine gastric mucins have been shown to prevent infection of epithelial cells by a variety of viruses including human papilloma virus type 16, Merkel cell polyoma-virus and a strain of influenza A virus (Lieleg et al., 2012). Furthermore, the vaginal microbiota composition also appears to impact the cervicovaginal mucus. A *Lactobacillus crispatus*-dominant microbiota has been shown to impede the diffusion of HIV-1 virions, in contrast to the rapid diffusion observed with a *Lactobacillus iners*-dominant microbiota, or when Gardnerella vaginalis is present in significant amounts (Nunn et al., 2015).

The impact of the gut microbiome on GI barrier permeability, and specifically its association with hyperpermeability, colloquially referred to as the “leaky gut,” has been extensively studied in multiple disease models (Nagpal and Yadav, 2017). Regulation of tight junctional proteins, which are multiprotein complexes controlling paracellular transport between epithelial cells, by the GI microbiota is a well-documented mechanism in this instance (Al-Asmakh and Hedin, 2015). Furthermore, some species, including *Lacticaseibacillus casei* and *Bifidobacterium adolescentis*, have been found to indirectly maintain barrier permeability by producing metabolites associated with reduced expression of the rotavirus toxin NSP4, which acts to disrupt the structure and function of tight junctions during infection (Gonzalez-Ochoa et al., 2017). Although the translation of *in vitro* and animal study outcomes to human populations is challenging, a variety of studies do suggest that probiotics are able to improve mucosal barrier function (Bron et al., 2017).

Antimicrobial peptides are defensive compounds common to practically all life forms, spanning from bacteria to humans (Jenssen et al., 2006). Bacterial AMPs, also known as bacteriocins, are produced by all major lineages of bacteria and have traditionally been considered a key probiotic trait (Dobson et al., 2012). The antimicrobial activity of bacteriocins against bacterial pathogens is well documented (Dobson et al., 2012), however some may also have antiviral properties, although the mode of action has received far less attention. Two modes of action seem to emerge from available data. On the one side, some bacteriocins seem to display antiviral activity before viral entry into human cells. In this regard, duramycin, a class-1 bacteriocin produced by *Streptomycetes*, has been found to prevent Zika virus entry by blocking its co-receptor TIM1 (Tabata et al., 2016). On the other side, several bacteriocins do not prevent viral entry, yet reduce cytopathic effects and viral release yield by interfering with late steps of the viral cycle. For instance, a 5kDa bacteriocin of *Lactobacillus delbrueckii* has no effect on early steps of influenza virus infection, but reduces production of viral proteins in infected cells (Serkedjieva et al., 2000). Similarly, the bacteriocin subtilosin, produced by species of the *Bacillus* genus, disrupts late infectious stages of both HSV type 1 (Torres et al., 2013) and HSV type 2 (Quintana et al., 2014). In particular, virus replication up to protein synthesis stage is not affected, but the intracellular localization of viral glycoprotein gD is altered. Conversely, enterocin CRL35 from Enterococcus faecium was found to lower glycoprotein gD synthesis, (Wachsman et al., 2003) thus seemingly disrupting a slightly earlier step in the infectious cycle of HSV compared to subtilosin.

The first stage of viral infection involves attachment of the virion to the host cell (Doms, 2016). Strains of *Lactobacillus (sensu lato)* and *Bifidobacterium* have been shown, in a cell culture model, to interfere with attachment and entry of vesicular stomatitis virus into cells, possibly by steric hindrance (Botić et al., 2007). Furthermore, *Lactobacillus* spp. (*sensu lato*) expressing CD4 receptors in their cell walls may be capable of binding and capturing the HIV-1 pseudovirus, thereby preventing attachment and reducing viral infection of CD4+ cells (Su et al., 2013).

The innate immune system refers to nonspecific, immediate defense mechanisms responding to viral antigen exposure. This system comprises physical barriers (e.g., epithelial cell surfaces), cellular receptors (e.g., toll like receptors (TLR)), anti-microbial peptides (e.g., defensins), and innate leucocytes (e.g., natural killer cells (NK) and phagocytes). Epithelial cells, as well as macrophages and dendritic cells (DCs), continually sample the mucosal environment and detect the presence of invading viruses through pattern-recognition receptors (PRRs) (Holt and Strickland,). Plasmacytoid dendritic cells (pDC) are specialized immune cells that recognize both viruses and bacteria, and they play an important role in inducing the cytotoxic activity of NK cells *via* the production of interferon-alpha (IFN-a) - interferons are cytokines that inhibit viral replication by interfering with the transcription of viral nucleic acid. In a pre-clinical study, phagocytosis of the strain *Lactococcus lactis* JCM 5805 by pDCs significantly stimulated IFN production *via* TLR9/MyD88 signaling (Jounai et al., 2012). In humans, NK cells are recognized to play a critical role in viral infection. A recent review of six randomized, placebo controlled clinical trials (RCT), with various strains and dosages, concluded that probiotics significantly increased NK cell activity in healthy elderly individuals (Gui et al., 2020). The authors did however caution that this conclusion is not definitive given the heterogeneity among studies and small numbers of participants involved.

Adaptive immunity refers to delayed but highly pathogen-specific defense mechanisms, like antibody-producing B-cells and T-cells of types CD4 (helper) and CD8 (cytotoxic), which have the capacity to produce pathogen-specific memory. The probiotic strain *L. rhamnosus* GG (ATCC53103), for example, produces the soluble factor Msp2 (or p40) protein, which signals epithelial cells in the gut to stimulate B-cells into producing IgA antibodies (Wang Y. et al., 2017). Indeed, probiotic strains, including LGG, have been shown to increase IgA in animal models (Wang Y. et al., 2017), and virus neutralizing serum immunoglobulins in humans when administered as adjuncts to some viral vaccines (De Vrese et al., 2005).

## The Microbiome, Dysbiosis, and Viral Infections

Dysbiosis has been broadly defined as any change to the composition of resident commensal communities relative to those found in healthy individuals (Petersen and Round, 2014). This alteration is thought to disrupt the symbiosis between host and microbes with potentially deleterious consequences. What a healthy human gut microbiome constitutes is a question without a simple answer, and one that has been recently addressed by a working group of the International Life Sciences Institute North America (Mcburney et al., 2019). Some of the challenges include the high degree of intra- and interindividual variation seen in human microbiomes (those of identical twins, for instance, are barely more similar than those of fraternal twins (Turnbaugh et al., 2010)), a lack of validated biomarkers to define and measure microbiome-host interactions, and the fact that it remains to be established if dysbiosis is a cause, or consequence, of changes in human physiology and disease (Mcburney et al., 2019).

Dysbiosis is frequently described taxonomically – such as an increase in potential pathogenic species (e.g., Gammaproteobacteria), decrease in organisms considered to be beneficial (e.g., *Bifidobacterium*, *Akkermansia*, or *Faecalibacterium* species), or change in alpha diversity (richness and evenness of species in a given microbiome); high alpha diversity is a marker of health in the GI tract but conversely a marker of dysbiosis in the vaginal microbiome (Borgdorff et al., 2016). Dysbiosis can also be described in functional terms such as the production of deleterious microbially derived compounds (e.g., hydrogen sulfide produced by sulfate reducing bacteria) (Levy et al., 2017). In fact ecosystem functionality is likely more important than the presence or absence of specific residents of the microbiome (Mcburney et al., 2019).

Numerous lines of evidence demonstrate that variation in microbiome structure and function impacts on viral immunity. This section will explore a number of key topics relevant to the dysbiosis-viral immunity relationship including: early life factors, antibiotic exposure, immunosenescence and inflammaging, diet and inflammatory diseases.

### Interaction Between the Early Life Microbiome, Immune System, and Viral Infections

The impact of dysbiosis on the immune system is perhaps most critical during the first 1,000 days of life; the time between conception and the child’s second birthday (Robertson et al., 2019). The microbiome is highly dynamic during this period of life due to the influence of several host-related and environmental factors including delivery mode, diet and antibiotic exposure (Tamburini et al., 2016). During early life, there is a process of ecological installation with a succession of species which leads finally to the establishment of a relatively stable community. Variations in these early interactions appear to set the tone of the mucosal and systemic immune systems into childhood and even adult life (Belkaid and Hand, 2014).

The most significant initial microbial inoculation of the neonate occurs in the peripartum period and varies depending on mode of delivery. Infants delivered by cesarean section (CS) tend to be colonized by common skin (e.g., *Staphylococcus, Streptococcus*, *or Propionibacteria*) *(* Tamburini et al., 2016) and opportunistic pathogens associated with the hospital environment (e.g., *Enterococcus, Enterobacter*, and *Klebsiella* species) (Shao et al., 2019), in contrast to the initial abundance of *Lactobacillus* spp. (*sensu lato*) seen in infants delivered vaginally. The extent of these different colonization patterns become less pronounced with time, however the diversity of the Bacteroidetes phylum, and microbiota diversity overall, is lower in CS delivered infants during the first 2 years of life (Jakobsson et al., 2014). This initial difference may have important consequences for viral immunity considering that bacterial colonization is necessary for the development and regulation of the immune system. Epidemiological studies have shown an association between CS delivery and chronic immune disorders, such as asthma (Sevelsted et al., 2015), which are associated with an increased risk of viral infections (Juhn, 2014). Thus the relationship between mode of delivery, initial bacterial colonization and aberrant immune function, if causal, suggests the potential of early preventative interventions. Although early life treatment with probiotics have demonstrated a benefit in reducing the incidence of some atopic diseases, such as atopic dermatitis (Li L. et al., 2019), the results in asthma, to date, have been less impressive (Wei et al., 2020).

Early feeding has a major influence on the microbiota composition, as breast-fed infants have consistently greater levels of bifidobacteria and an overall lower diversity compared to formula-fed infants (Milani et al., 2017). Human milk is considered to be the best source of nutrition for most newborns and infants. It is a complex fluid, containing a diverse array of microorganisms, carbohydrates and bioactive components (e.g., IgA). Some of the bacterial species found in human breast milk, such as *Lactobacillus* spp. (*sensu lato*) and *Bifidobacterium* spp., may transfer from the maternal gut *via* an entero-mammary pathway, a recently discovered form of mother-neonate communication, and are thought to protect against many respiratory and GI infections (Le Doare et al., 2018). Breast milk also contains >200 structurally different human milk oligosaccharides (HMOs) (Jost et al., 2015), which cannot be digested by the infant, but rather promote the growth of selected genera, such as Bifidobacteria and Bacteroides (Marcobal et al., 2010), thought to promote the healthy development of the GI tract and immune system. HMOs are only partially digested in the small intestine and mostly reach the colon, where they are fermented to produce short-chain fatty acids (SCFA) and lactate (Yu et al., 2013). These metabolites create an acidic pH which is known to inactivate some common enteric viruses (Al Kassaa et al., 2014), while the infant immune system matures. This pH-depending microbiota effect illustrated the evidence on the role of microbiota suppressing viral infectivity and its role as a “co-helper” of the infant immune system (Li N. et al., 2019).

A recent prospective study of long-term associations of breastfeeding and infections among 6-year-old children in the United States suggests that breastfeeding may protect against ear, throat, and sinus infections well beyond infancy (Li et al., 2014). Evidence also suggests that breastfeeding reduces the odds of childhood obesity (Weng et al., 2012), which is known to increase the risk of adulthood obesity; itself a risk factor for some viral infections (Milner and Beck, 2012). A possible mechanistic explanation of the interconnection between breast-feeding, the microbiome and viral immunity was recently identified in infant rhesus macaques (Ardeshir et al., 2014). Community richness, evenness and diversity was greater in the microbiota of breast-fed compared to formula-fed animals, features that were positively correlated with T_H_1, cytotoxic T, and T_H_17 cells. Furthermore, breast-fed infants had a significantly greater abundance of *Prevotella* spp. and arachidonic acid. *Prevotella* spp. express phospholipase A2 which is able to release arachidonic acid from phospholipid membranes. This finding is of particular relevance to viral infections as human infants receiving greater amounts of arachidonic acid were at lower risk of contracting HIV from their HIV+ mothers, and enhanced T_H_17 populations have been associated with reduced viral loads associated with infection from simian immunodeficiency virus (Ardeshir et al., 2014). Another really recent illustration of this significant role of the microbiota during infant period is an investigation of the link between microbiota composition and the response to rotavirus vaccine Rotarix (Harris et al., 2017). This study analyzed the serum IgA antibody responses to Rotarix^®^ and fecal microbiota in 78 Ghanaian infants and compared the resulting 39 responder and non-responder Ghanaian pairs to Dutch infants. The overall microbiome composition was significantly different between responders and non-responders to vaccination, with the Ghanaian responders being more similar to healthy Dutch infants than non-responders. Responses to Rotarix^®^ correlated with an increased abundance of *Streptococcus bovis* and the lack of response is correlated with the high abundance of *Bacteroidetes* phylum, especially several bacteria related to species from the *Bacteroides* and *Prevotella* genera. This study highlighted the correlation of the gut microbiota composition with the seroconversion rate in infants after vaccination. Finally, the microbiota has come to the forefront of infection and immunity research, as there are mounting evidence suggesting that microbiota plays an important role in the development of host immune system and immunity especially in the first years of life.

### Antibiotic-Induced Dysbiosis and Viral Immunity

Numerous epidemiological studies suggest that early life antibiotic exposure may increase the risk of developing immune related illnesses in later life, such as asthma, eczema and food allergies (Tamburini et al., 2016). Early life antibiotic exposure in humans also appears to impair antiviral immunity, however this relationship may not be causal but rather explained by genetic variants impacting antiviral immunity (Semic-Jusufagic et al., 2014). Studies using gnotobiotic (germ-free), antibiotic-treated, mice have demonstrated that manipulation of commensal bacteria causes impaired lymphoid tissue development, dysregulated immune cell homeostasis, and altered susceptibility to infectious and inflammatory diseases in the GI tract (Abt et al., 2012). In a rodent model antibiotic administration to mothers during pregnancy and lactation caused substantial alterations to theirs and their offspring’s GI microbiota (Gonzalez-Perez et al., 2016). The infant mice in this study experienced increased and accelerated mortality following viral infection. Antigen specific IFN-y–producing cytotoxic CD8+ T cells were found to be reduced in sub-lethally infected infant mice. Furthermore, CD8+ T cells from uninfected infant mice also demonstrated a reduced capacity to sustain IFN-y (a critical cytokine for antiviral defense) production following *in vitro* activation (Gonzalez-Perez et al., 2016). Furthermore, mice treated with antibiotics exhibit impaired innate and adaptive antiviral immune responses, and substantially delayed viral clearance after exposure to systemic lymphocytic choriomeningitis virus or mucosal influenza virus (Abt et al., 2012). This is because commensal bacteria appear to provide tonic signals that calibrate the activation threshold and sensitivity of the innate antiviral immune system. Bradley et al. demonstrated that the GI microbiota signals to lung stromal cells to keep them in an IFN-primed state thus affording them protection from influenza virus infection through early blunting of the viral life cycle (Bradley et al., 2019). Oral antibiotic treatment in this study was shown to impair this antiviral mechanism in rodents and thus increase susceptibility to viral infections. Intriguingly, this antibiotic induced impairment was shown to be reversed with fecal transplantation from control mice (Bradley et al., 2019). In a further study, dysbiosis, caused by oral antibiotic treatment, directly impaired antiviral immunity following mucosal herpes simplex virus type 2 infection of the vaginal mucosa (Oh et al., 2016). The authors reported that depletion of commensal bacteria was associated with secretion of IL-33 from the vaginal epithelium, which suppressed local antiviral immunity by blocking the migration of effector T cells to the vaginal tissue, thereby inhibiting the production of IFN-γ.

### Age-Related Dysbiosis and Viral Immunity

As we age many of our body functions will gradually be reduced. This goes hand-in-hand with changes in health status, lifestyle and diet. In addition to the more obvious reductions in motility and cognition, there are also changes in the composition and activity of the intestinal microbiota and associated immune responses. Besides the often-mentioned reduction in fecal bifidobacteria, other changes in the composition of the fecal microbiota have been observed. A reduction in butyrate producing organisms from the Clostridium cluster XIVa has been reported as well as a reduction in anti-inflammatory organisms such as *Faecalibacterium prausnitzii* and *Akkermansia muciniphila* (Mangiola et al., 2018). This is important given that the maintenance of the fecal microbial diversity is considered beneficial for healthy aging (Kim and Jazwinski, 2018). The decline in immune function associated with aging, immunosenescence, leads to impaired innate immunity, with reduced Natural Killer (NK) cell and phagocytic activity. This may lead to a reduced response to viral infections and vaccinations (Przemska-Kosicka et al., 2018). Probiotics have been reported to improve the NK and phagocytic responses in the elderly (Miller et al., 2019).

Despite the decrease in some aspects of the immune system characteristic of immunosenescence, there is also a heightened pro-inflammatory status in older individuals; often referred to as “inflammaging.” (Kim and Jazwinski, 2018) The GI microbiome is thought to be a key player in this process as it is essential for the proper maintenance of the intestinal barrier through various mechanisms, e.g., production of SCFA (Bron et al., 2017) as well as tryptophan metabolites (Rothhammer and Quintana, 2019). A reduced intestinal barrier function is associated with the translocation of pro-inflammatory, bacterially derived products, such as lipopolysaccharides (LPS), into the body (Mangiola et al., 2018). This in turn is associated with increased levels of circulating inflammatory markers such as interleukin (IL)-1, IL-1 receptor antagonist protein (IL-1RN), IL-6, IL-8, IL-13, IL-18, C-reactive protein (CRP) and tumor necrosis factor (TNF)-α, and immune regulating cytokines such as transforming growth factor (TGF)-β (Ctoi et al., 2020). This chronic low level inflammation exacerbates, or maintains, the hyperpermeability of the intestinal barrier, which combined with increased production of reactive oxygen species (ROS), contributes to diminished T-cell function (Pinti et al., 2016) and thus possible impairment in viral clearance during infection. There is compelling evidence that probiotics are able to support the intestinal barrier (Bron et al., 2017), which may act to dampen the increased inflammatory tone seen in aging and enhance the immune response to viral infections and vaccinations. However, it remains to be definitively proven that such interventions are clinically efficacious (Aiello et al., 2019).

### Diet-Related Dysbiosis and Viral Immunity

Our divergence from chimpanzees some six million years ago has been accompanied by a substantial loss of gut bacterial diversity (Moeller, 2017). Early dietary shift away from plant-based foods toward animal tissues likely initiated this bacterial depauperation, however this has been dramatically accelerated in the last ~200 years (i.e., since the Industrial Revolution) with the widespread introduction of refined flour and sugar into our diets (Adler et al., 2013). As the GI microbiome is deeply integrated with the mammalian hosts’ immune system, rapid and substantial contractions in diversity, a consequence of modern lifestyles, may increase the risk of infections and immune related diseases (Moeller, 2017). Diet is known to have a substantial short and long term impact on the microbiome profile (David et al., 2014). Human research comparing the microbiomes between children from rural Africa and urban Europe discovered that the former was characterized by: I) higher microbial richness and biodiversity, II) significant enrichment in Bacteroidetes and depletion in Firmicutes, III) significantly less pathogenic *Enterobacteriaceae* (*Shigella* and *Escherichia*) and IV) specific abundance of bacteria from the genus *Prevotella* and *Xylanibacter*, which contain genes for dietary fiber digestion; unsurprisingly associated with significantly more short-chain fatty acids (De Filippo et al., 2010). Western diets, high in processed foods and animal fats, and low in plant-derived elements, have been shown to alter the microbiome, promote inflammation and deleteriously affect immunity with important implications to health (Tilg and Moschen, 2015). Specifically, a lack of fermentable fiber in the diet results in reduced microbiota-driven production of SCFAs, which are known to play an important role in both adaptive and innate immunity (Krautkramer et al., 2016). In mice, depletion of acetate-producing Bifidobacteria increased susceptibility to infections and promoted GI inflammation (Tilg and Moschen, 2015), and a high-fat diet, independent of obesity, altered the composition of the intestinal microbiome (Hildebrandt, 2009). Moreover, in a rodent model, depriving animals of enteral nutrition (e.g., parenteral nutrition – intravenous nourishment) significantly alters intestinal bacterial colonization (e.g., increases levels of Proteobacteria (Demehri et al., 2015)) and causes defects in specific mucosal immunity (e.g., lowered secretory IgA levels). These changes are associated with increased intestinal bacterial translocation and the loss of respiratory antiviral defenses in mice (Renegar et al., 2001). Thus alterations in dietary fiber, and resultant SCFA profiles, as well as the route of energy provision impacts on the microbiome and may potentially influence viral immunity.

### Disease-Related Dysbiosis and Viral Immunity

Disruption in human–microbiome symbiosis is associated with, and may contribute to, the increasing prevalence of numerous chronic diseases including inflammatory bowel disease (IBD), obesity, rheumatoid arthritis (RA) and asthma (Lloyd-Price et al., 2016). These diseases feature significant microbiome alterations, which are thought to play a role in inducing the consistent hallmarks of local and systemic inflammation (Hand et al., 2016). Perhaps unsurprisingly the risk of viral infections may be greater in these chronic inflammatory diseases, as recently highlighted by the COVID-19 pandemic and obesity (Finer et al., 2020). Furthermore, chronic diseases, such as RA, characterized by immune dysfunction and dysbiosis, are of great clinical and mechanistic interest as they provide insights into the pathophysiology of viral infections (Schett et al., 2020). The central role of the gut microbiome in viral immunity is highlighted in human vaccination studies in a dysbiosis context: gut microbiome modulation by antibiotics has been showed to modify immune response to Rotavirus vaccination (Harris et al., 2017). For Influenza, antibiotic-induced gut dysbiosis impaired response to H1N1 vaccination (Hagan et al., 2019) with enhanced inflammation. This may be linked to microbiota sensing through TLR5 (Oh et al., 2014). Interestingly, variations in TLR5 genes are associated with a number of inflammatory diseases (Leifer et al., 2014).

A number of chronic inflammatory diseases are discussed here to further illustrate possible links between inflammation, the microbiome and viral infections.

#### Inflammatory Bowel Diseases (IBD)

IBD patients, either ulcerative colitis (UC) or Crohn’s disease (CD), are at higher risk of viral infections and therefore require specific antiviral care including sero-surveillance, vaccination and anti-infective therapies. This increased risk is due to immunological impairment induced by both the pathophysiology of the disease and immuno-modulating medications (Marín et al., 2015; Magro et al., 2017). This is thought to influence the reduced immunogenicity of HBV and influenza vaccines in IBD patients, for which the underlying pathomechanism remains to be elucidated (Marín et al., 2015). Dysregulated immune-microbiota dynamics likely play a substantial role in the multifactorial etiology of IBD. The microbiomes of IBD patients tend to display greater instability and reduced diversity compared to healthy subjects (Halfvarson et al., 2017). The presence of some pathogenic microbes (e.g., invasive *Escherichia coli*) activate T helper 1 and T helper 17 cells thereby inducing mucosal damage (Sartor and Wu, 2017). This results in increased uptake of microbially-derived antigens and the translocation of viable gut micro-organisms, which continue to promote a dysregulated immune response. The GI metagenomes of IBD patients are also markedly different from non-IBD controls, showing an increased tolerance for oxidative stress and a correlation with inflammatory markers (Franzosa et al., 2019). Probiotics may provide an option to mitigate some microbiome-related effects in IBD and to help restore a normal immune function by acting on the intestinal gut barrier integrity for example (Sartor and Wu, 2017). Current clinical research findings suggest a role for probiotics in pouchitis and mild/moderate UC but not in CD (Abraham and Quigley, 2017). A recent meta-analysis called for more definitive research in UC (Kaur et al., 2020).

#### Obesity

Numerous studies have reported obesity to be a predictor of worse outcomes from infection with the 2009 influenza A (H1N1) pandemic strain (Milner and Beck, 2012). Obesity has also been linked with longer influenza A virus shedding (Maier et al., 2018) and more severe forms of COVID-19 infections (Stefan et al., 2020). Furthermore, evidence suggests that obesity is associated with an impaired response to vaccination (Milner and Beck, 2012). Cross-sectional studies have shown that certain bacterial populations—such as *A. muciniphila*, *F. prausnitzii*, *Methanobrevibacter smithii* and *Christensenellaceae*—are better represented in lean individuals compared to those who are overweight or metabolically unhealthy (Stenman et al., 2016). A wealth of research has identified that the dysbiosis seen in obesity is associated with a low grade systemic inflammation (Hartstra et al., 2015), which may be responsible for impaired viral immunity. Interestingly, a meta-analysis showed that probiotic strains from specific taxa (*Bifidobacterium breve, B. longum Streptococcus thermophilus Lactobacillus acidophilus, L. delbrueckii*, and *Lacticaseibacillus casei*) may positively impact anthropometric outcomes and metabolic risk factors such as fasting glucose or insulin in individuals with metabolic diseases (Koutnikova et al., 2019). Specific mechanisms mediated in the gut by the ingested probiotics, such as sugar digestion and absorption or increased production of short chain fatty acids, might be involved (Panwar et al., 2014; Stenman et al., 2016).

#### Asthma

Asthma is in part initiated by recurrent viral infections during early childhood. Asthma features deficient antiviral immunity and exacerbations are elicited by respiratory viruses (Altman et al., 2020). As a result asthma patients are believed to be more susceptible to contracting SARS-CoV-2, and have an increased risk of severe infection. However, it is currently unclear if asthma is indeed a risk factor for COVID-19 complications (Hosoki et al., 2020), and studies so far have not conclusively observed the expected increase in incidence. The Th-2 cytokine profile of asthma or concomitant therapies might explain this apparent contradiction (Liu S et al., 2020). Consistent with the associations found in obese individuals, levels of *A. Muciniphila* and *F. Prausnitzii* are reduced in the gut microbiota of children with allergic asthma compared to healthy controls (Demirci et al., 2019). The available evidence from human intervention studies is limited to early childhood, where, for example, synbiotics have shown positive effects in asthma associated with an increase in bifidobacteria (Wopereis et al., 2014; Zhao et al., 2019).

#### Rheumatoid Arthritis (RA)

RA patients are at increased risk of infection compared to the general population owed to disease related impairment in immune system function and immunosuppressive drugs (Listing et al., 2013; Favalli et al., 2020). Regarding viral infections specifically, studies indicate an increased risk of infection from viral upper respiratory tract pathogens, herpes simplex and herpes zoster viruses (Widdifield et al., 2013; Arleevskaya et al., 2017). Metagenomic shotgun sequencing and metagenome-wide association analysis of the oral and fecal microbiomes of RA patients have identified enrichment in gram positive bacteria and depletion in gram negative bacteria compared to healthy controls, however, the overall richness and diversity were comparable between groups (Zhang et al., 2015). Disease-modifying antirheumatic drugs (e.g., methotrexate) appear to partially restore microbiome features toward those of healthy controls (Zhang et al., 2015). A recent meta-analysis of four probiotic trials in RA identified some improvement, however, due to a scarcity in data, further definitive research is needed (Aqaeinezhad Rudbane et al., 2018).

Overall, while there is an increased risk of viral infection in certain chronic inflammatory disorders, this association is not systematic and varies with disease and viral pathogen. Patterns of dysbiosis also vary between different inflammatory diseases and the mechanisms linking these microbiome features to viral infection susceptibility are currently unknown. Nevertheless, interventions aimed at maintaining or recovering both gut and lung microbiomes, in an inflammatory context (e.g., such as probiotics, prebiotics and synbiotics) are rational and may indirectly help to enhance the immune response to both acute and future viral infections.

## Probiotics and Viral Infections

Probiotics are most commonly bacterial species from the *Lactobacillus* (*sensu lato*) and *Bifidobacterium* genera. Such organisms are often used as treatments for conditions with an inflammatory component (e.g., infectious diarrhea, atopic dermatitis, IBD, migraine), however their true functionality is more nuanced, as they have been shown to induce both pro- (Chiba et al., 2010) and anti-inflammatory (Plaza-diaz et al., 2013) responses. Therapies that exert their effects *via* the microbiome (e.g., probiotics, prebiotics and postbiotics; non-viable microbial cells, microbial fractions, or cell lysates (Aguilar-Toalá et al., 2018)) are widely available and have a long history of use for many different diagnoses.

This final section will explore the evidence for probiotics in a number of key viral infections including: viral gastroenteritis, viral hepatitis, human immunodeficiency virus (HIV), human papilloma virus (HPV), viral upper respiratory tract infections (URTI), influenza infection and vaccination, SARS CoV-2 infection and in relation to intensive hospital care.

The mechanisms by which selected strains of probiotics may elicit these antiviral activity activities are not fully understood. However, hypotheses for these mechanisms exist. Similar as the endogenous microbiota, probiotics may improve mucosal barrier function, limiting the ability of viral particles to cross this physical barrier and maintain this barrier during viral disease (Bron et al., 2017). Direct virucidal effects have been observed for selected probiotic lactobacilli, primary metabolites such as hydrogen peroxide or lowering the pH by organic acids have such effects (Zabihollahi et al., 2012). Also, some bacteriocins have been observed to display antiviral activity, in addition to their well-known antibacterial activity (Torres et al., 2013; Quintana et al., 2014). Adhesion of the virus to the mucosal surface is the first step of infection. Exopolysaccharides produced by lactic acid bacteria have been shown to effectively interfere with this step of, e.g., adenovirus (Biliavska et al., 2019). Also other metabolites and bacterial cell fragments may interfere with virus binding (Martín et al., 2010) and *Levilactobacillus brevis* cell wall fragments have been reported to inhibit herpes simplex replication (Mastromarino et al., 2011). The most well documented anti-viral effect of probiotics, however, is the modulation of the immune system. Selected probiotics have been reported to increase natural killer cell activity and cytotoxic activity (Miller et al., 2019). Also antiviral cytokine responses such as IFN-γ, IL-2, IL-12, and IL-18 as well improved antibody responses have been reported (Lehtoranta et al., 2020). It is not unlikely that that many of these and other mechanisms function in parallel and/or subsequently.

### Viral Gastroenteritis and Probiotics

Over 20 viruses, bacteria and parasites are known to cause acute gastroenteritis. Worldwide, rotavirus is the most common cause of gastroenteritis and over 450,000 deaths each year are attributed to rotavirus infection (Allen et al., 2010; Gonzalez-Ochoa et al., 2017). Astrovirus, norovirus, sapovirus and adenovirus are other important causes of acute viral gastroenteritis.

The rotavirus proteins NSP1 and NSP4 have been shown to be important in the pathogenesis of rotavirus gastroenteritis (Gonzalez-Ochoa et al., 2017). NSP1 has been shown to inhibit INF production, leading to down-regulation of inflammatory cytokines (Gonzalez-Ochoa et al., 2017). NSP4 protein is a viral toxin which affects the structure of the intestinal mucosa (targeting tight junctional complexes) and affecting the relative intra- and extra-cellular electrolytes balance and ultimately result in secretory diarrhea. Interestingly, there is evidence that probiotics may lead to decreased viral shedding by impeding rotavirus attachment, penetration and replication within enterocytes (Rigo-Adrover et al., 2017). Recent research has also shown that metabolites of *Lacticaseibacillus casei* and *Bifidobacterium adolescentis* were associated with reduced expression of the viral toxin NSP4 (Gonzalez-Ochoa et al., 2017).

Conventional management of rotavirus gastroenteritis consists of oral rehydration solutions (ORS) to replace fluids and electrolytes lost by vomiting and diarrhea. Zinc supplements are also recommended by the World Health Organization, as this has been shown to improve oral rehydration. Despite the first rotavirus vaccine being licensed in 1998, there is yet to be widespread global uptake (Offit, 2018). Universal access to the rotavirus vaccine will hopefully reduce the global significance of this virus, however, until then, the importance of effective treatment strategies to public health will remain.

Probiotics have a long history of use in diarrheal diseases and there are many well designed RCTs to establish their effectiveness in the management of infective diarrheal disease. In 2010 the Cochrane group published a systematic review and meta-analysis of 63 RCTs, including a total of 8,014 participants (Allen et al., 2010). This wide-reaching analysis, including multiple different causative organisms and multiple different types of probiotic treatments, provides an extremely useful overview of probiotics for acute gastroenteritis. This meta-analysis demonstrated a clear, statistically significant reduction in diarrheal symptoms: probiotics reduced the mean duration of diarrheal symptoms by 24.76 h (95%CI 15.9 – 33.6 h) when compared to placebo. Furthermore, a 2016 review concluded that selected probiotic strains decreased both the duration of diarrhea and hospitalization by approximately 24 h (Vandenplas, 2016). Meta-analyses such as these have some important limitations including the inherent weakness of summative conclusions based on significantly heterogenous formulations and often unconfirmed, and thus possibly variable, responsible pathogens. However, despite these cautions, probiotics are beginning to move into the mainstream of treatment guidelines. The European Society for Pediatric Gastroenterology, Hepatology and Nutrition (ESPGHAN) published guidance in 2020 that certain probiotic strains (e.g., *Saccharomyces boulardii* and *L. rhamnosus GG*), with a clear evidence base from multiple clinical trials, could be safely used as adjunctive treatment for acute gastroenteritis in previously healthy infants and children (Szajewska et al., 2020). Added to this, the Canadian Pediatric Society recommends using probiotics to shorten the duration of symptoms in cases of viral gastroenteritis (Marchand et al., 2012). In contrast, the American Gastroenterological Association has recently advised against such use, arguing that most of the studies documenting the effect of probiotics on acute gastroenteritis in infants were done outside of North-America (Su G. et al., 2020). Probiotic clinical research in the management of acute gastroenteritis is relatively advanced compared to other indications, however, it is clear that further definitive evidence is needed to understand the most effective strains, or combination of strains, and dosages.

### Viral Hepatitis and Probiotics

Hepatitis B (HepB) infection is a worldwide healthcare problem, especially in developing countries. Pathogenic interaction between the virus and host immune system leads to liver injury and, potentially, cirrhosis and hepatocellular carcinoma (Wang J. et al., 2017). GI microbiota dysbiosis, through the gut-liver axis (Milosevic et al., 2019), has been shown to drive the progression toward severe forms of liver failure, including inflammation and pathogenic metabolite accumulation associated with chronic hepatitis B (CHB) (Wang J. et al., 2017). The dysbiosis associated with HepB infection appears to involve a gain in potentially pathogenic bacteria (e.g., some Enterobacteriaceae) and a loss in potentially beneficial bacteria; namely *Bifidobacterium* species (Zeng et al., 2020). The protective mechanisms offered by bifidobacteria include reduction in endotoxin levels (GI and plasma), compositional modulation of gut microbiota, antimicrobial production, enhanced GI barrier function and modulation of local and systemic immunity (Zeng et al., 2020). This situation offers possible therapeutic strategies for monitoring and altering gut dysbiosis in CHB patients. In an *in vitro* cellular model (HepG2.2.15 - contains integrated HBV DNA and secretes HBsAg) cell extract of *B. adolescentis* SPM0212 inhibits HBV, and its antiviral mechanism is associated with the Mx GTPase pathway; one of the four main effector pathways of the IFN-mediated antiviral response (Lee D. K. et al., 2013). Furthermore, in a study published in 2010 infants whose HepB vaccination schedule (two doses monovalent + one dose combination) was supplemented with probiotics (during 1^st^ 6 months of life) demonstrated enhanced HepB virus surface antibody (HBsAb) immunoglobulin G production vs placebo (Soh et al., 2010).

Similar to the case in CHB the gut microbiota also appears to be associated with the progression of chronic Hepatitis C (CHC) and related complications (Inoue et al., 2018). GI bacterial alpha diversity significantly decreases in CHC patients compared with healthy individuals and is associated with the severity of the clinical stage (Inoue et al., 2018). Interestingly a specific species, Streptococcus salivarius, was found to be drastically increased in association with CHC progression (Inoue et al., 2018).

The gut microbiome is known to metabolize and modulate the pharmacokinetics and pharmacodynamics of a number of pharmaceuticals. Interestingly, deleobuvir, an experimental non-nucleoside hepatitis C virus NS5B polymerase inhibitor (inhibitor of hepatitis C virus), showed different metabolic profiles following incubation with human liver microsomes compared to fecal homogenates suggesting bacterial modulation (McCabe et al., 2015).

Persistent infection with hepatitis B and C viruses causes liver injury leading to progressive fibrosis and liver cirrhosis. The progression of cirrhosis is associated with increased intestinal permeability, small intestinal bacterial overgrowth, and bacterial translocation. This situation permits microbially derived compounds (e.g., ethanol, acetaldehyde, lipopolysaccharide) to access the liver *via* the portal circulation stimulating production of pro-inflammatory cytokines that promote liver inflammation, fibrogenesis and ultimately cirrhosis (Wang et al., 2019). Hepatic encephalopathy (HE), characterized by personality changes, motor disturbance, and depressed level of consciousness, is a life threatening complication occurring in 30-45% of patients with liver cirrhosis. The exact underlying mechanisms of HE in patients with cirrhosis remains unclear, but hyperammonaemia and systemic inflammation are thought to play a critical role. Interestingly, research has shown a significant increase in *Alcaligenaceae*, *Porphyromonadaceae*, *Veillonellaceae*, *Enterococcus*, *Megasphaera*, and *Burkholderia* in the GI microbiomes of cirrhotic patients with HE. This taxonomic shift was associated with hyperammonaemia and systemic inflammation contributing to the exacerbation of HE symptoms (Sung et al., 2019). In an open-label, randomized controlled trial probiotics were found to be effective for the secondary prophylaxis of HE in patients with cirrhosis (36% viral hepatitis related) (Agrawal et al., 2012). Furthermore, in a prospective, randomized controlled trial, probiotics were also found to be effective in the primary prevention of HE in patients with cirrhosis (26.3% viral hepatitis related) (Lunia et al., 2014). Probiotics may act by replacing the pathogenic pro-inflammatory, urease-producing organisms thus preventing the development of HE.

### Human Immunodeficiency Virus, the Vaginal Microbiome and Probiotics

The healthy microbial composition of the vagina is characterized, unlike other mucosal niches, by a low microbial diversity and is often dominated by a single *Lactobacillus* (*sensu lato*) species. Advances in molecular technologies in recent years have revealed that the vaginal microbiomes of reproductive-aged women can be broadly categorized into five different community state types (CSTs) (Smith and Ravel, 2017). A key commonality is that CSTs fall into two clear groups: (i) *Lactobacillus* (*sensu lato*) dominant, where one or more species of *Lactobacillus* (*sensu lato*) make up >90% of the total copy number or sequencing reads (*L. crispatus* CST-I*, L. gasseri* CST-II*, L. iners CST-III and L. jensenii CST-V*), and (ii) non–*Lactobacillus* (*sensu lato*) dominant (CST-IV), in which *Lactobacillus* (*sensu lato*) makes up <30% of a polymicrobial mixture of strict and facultative anaerobes (e.g., species of the genera Gardnerella) and other taxa in the order Clostridiales (Smith and Ravel, 2017). Vaginal dysbiosis consists of a prolonged deviation from a low-diversity, *Lactobacillus* (*sensu lato*) dominated microbiome; bacterial vaginosis (BV), an anaerobic polymicrobial disease, associated with subclinical vaginal inflammation, is the most common type (De Seta et al., 2019). BV is generally treated with antibiotics (metronidazole or clindamycin), however, due to their inability to effectively penetrate biofilms, efficacy is often suboptimal and recurrence rates are high (Bradshaw and Brotman, 2015). Furthermore, prolonged antibiotic use increases the risk of side effects and drug resistance. A recent systematic review examined the impact of vaginally applied *Lactobacillus-*based probiotics (*sensu lato*) on the vaginal microbiome in 34 clinical studies (van de Wijgert and Verwijs, 2020). The authors concluded that probiotics hold promise for BV cure and prevention, however high heterogeneity and suboptimal quality of many studies prevented definitive conclusions. The mechanisms by which probiotics might improve BV include colonization resistance against pathogens, pro-inflammatory, biofilm forming bacteria (e.g., *Gardnerella vaginalis*), maintenance of healthy low pH environment (lactic acid production), increased antimicrobial compounds and modulation of cervicovaginal mucosal immunity (van de Wijgert and Verwijs, 2020). The state of the vaginal microbiome has an important relevance to human immunodeficiency virus (HIV). BV is associated with an increased risk of HIV infection in women, possibly by as much as 60% (Atashili et al., 2008), and increased HIV shedding (Bolton et al., 2008), therefore BV treatment may help to reduce HIV transmission by various means. Furthermore, the CAPRISA (Centre for the AIDS Program of Research in South Africa) 004 trial found that the antiretroviral drug, tenofovir (applied as a vaginal gel), reduced HIV acquisition by an estimated 39% overall (Karim, 2010). Interestingly, the researchers identified that tenofovir reduced HIV incidence by 61% (P = 0.013) in *Lactobacillus* (*sensu lato*) dominant women but only 18% (P = 0.644) in CST-IV women (Klatt et al., 2017). *G. vaginalis* and other anaerobic bacteria were found to metabolize tenofovir, and therefore deplete it, more rapidly than target cells converted it to its pharmacologically active state (Klatt et al., 2017). Taken together, these findings highlight the potential utility of assessing and modulating vaginal microbiota as a novel approach to reducing the transmission of HIV.

### Human Papilloma Virus, the Vaginal Microbiome and Probiotics

Human papillomavirus (HPV), a type of DNA virus, is estimated to infect up to 80% of sexually active women by age 50 (Moscicki, 2005). Although most HPV infections resolve over time, persistent infection can cause catastrophic cell-cycle instability and eventually lead to invasive cancer; mainly cervical intraepithelial neoplasia and cervical adenocarcinoma. Nevertheless, HPV presence alone is insufficient for cancer formation. Individual factors unique to the mucosal surface such as epithelial surface integrity, mucosal secretions, and immune regulation likely play a role in HPV persistence and progression to cancer (Pyeon et al., 2009; Schiffman et al., 2016). Further evidence indicates that the cervicovaginal microbiota plays a substantial role in the persistence and regression of the virus and thus has important implications for subsequent disease (Chang and Parsonnet, 2010; Mitra et al., 2016). A dysbiotic or high diversity vaginal microbiota coupled with chronic subclinical inflammation, hallmarks of BV, has been correlated with higher incidence, prevalence and persistence of HPV infection (Gillet et al., 2011; Guo et al., 2012). Recent observational cross-sectional studies support the concept that CSTs III and IV, in particular, are frequently linked with the presence of HPV infection and development of preinvasive cervical disease states (Lee J.E. et al., 2013; Brotman et al., 2014; Mitra et al., 2015).

It appears that through production of certain enzymes and metabolites, BV-associated microbes compromise the cervical epithelial barrier function that normally inhibits the entry of HPV to the basal keratinocytes (Borgdorff et al., 2016). Additionally, they act on several cellular pathways that engender persistent and productive viral infection (Hedges et al., 2006; Cheriyan et al., 2009). All of these bacterial, mucosal and immune complications related to BV can result in an increased susceptibility to HPV infection and the development of high-grade intraepithelial lesions. This is significant because it then follows that vaginal *Lactobacillus* spp. (*sensu lato*) limit colonization of BV-associated microbes and thus by extension are protective against the aforementioned deleterious viral effects through maintenance of a low pH (Breshears et al., 2015), and bacteriocin production (Stoyancheva et al., 2014). A deeper glance into the protective effects of the vaginal microbiota reveal that the type of *Lactobacillus* (*sensu lato*) profile proves pivotal. A *L. crispatus*-dominated vaginal microbiota produces high concentrations of D-lactate which has been recently shown to increase cervicovaginal mucus viscosity and enhance its viral particle trapping potential (Nunn et al., 2015), and tends to have the least likelihood of viral infections of all CSTs (Borgdorff et al., 2014).

Probiotics have been studied in the context of HPV with study outcomes aimed at enhanced genital viral clearance and quality of cervical smear. A prospective controlled study with 54 women with HPV and low grade squamous intraepithelial lesion diagnosis in their PAP smear were followed for 6 months (Verhoeven et al., 2013). After daily consumption of a probiotic drink, the intervention group had a twice as high chance of clearance of cytological abnormalities compared to the control group (60 vs. 31%, P=0.05). HPV was cleared in 19% of control patients versus 29% of probiotic users (P=0.41). The 3-month application of two probiotic strains, *Lacticaseibacillus rhamnosus* GR-1 and *Limosilactobacillus reuteri* RC-14, in a double-blind RCT involving 121 women with genital high-risk HPV (HR-HPV) infection did not influence genital HR-HPV clearance, but was able to decrease the rates of mildly abnormal and unsatisfactory cervical smears (P= 0.017) (Ou et al., 2019). Interestingly, long-term addition of vaginal probiotics has been demonstrated to have a superior ability to reduce cytological anomalies (P=0.041) and enhance HPV clearance (P=0.044) compared to short-term probiotic administration (Palma et al., 2018). Regardless of the approach or duration of treatment, it appears that the functional support of probiotics and eventual re-establishment of a protective vaginal microbiota are critical elements to successfully combat HPV infections.

### Viral Upper Respiratory Tract Infections and Probiotics

The upper respiratory tract is composed of the nostrils, nasal cavity, oral cavity, tonsils, pharynx and larynx. Upper respiratory tract infection (URTI) is an umbrella term that encompasses a range of pathogenic insults affecting these structures, e.g., the common cold (primarily affecting the nose), tonsillitis, pharyngitis, laryngitis, acute otitis media (middle ear infection) and sinusitis. Viral URTIs are most frequently caused by rhinoviruses, respiratory syncytial virus, adenoviruses, coronaviruses, influenza viruses, para-influenza viruses and human metapneumovirus (Bosch et al., 2013). Acute URTIs are the most common reason for people to seek medical care in the United States and the world (Cherry et al., 2003). Approximately one billion common colds occur each year in the US, with an average of two to six episodes per person per year. URTIs account for up to 75% of all antibiotic use in some countries, which, considering that such infections are mostly viral in origin, highlights the startling misuse of these precious medications and the scale of just one problem fueling the crisis of antibiotic resistance (Fendrick et al., 2001; Hao et al., 2015).

A significant number of clinical trials have examined the efficacy of probiotics to prevent URTIs. Broadly speaking probiotics are thought to prevent respiratory tract infections in similar ways to the prevention of GI infections - through modulation of local and systemic immunity. Specific effects may include enhancing phagocytic activity of peripheral leucocytes, increasing secretion of immunoglobulins (IgA, IgG, and IgM) and increasing production of cytokines (e.g., interleukins, TNF-α and interferon-α) (Hao et al., 2015). Future studies should aim to achieve a clearer understanding of the underlying mechanism(s) of action of specific probiotic strains, and strain mixes, responsible for the improvements seen in clinical endpoints (Ozen et al., 2015).

A 2015 Cochrane review examined 12 probiotic RCTs for the prevention URTI, involving a total of 3,720 participants (Hao et al., 2015). The trials included data from all age groups - children, adults, and older people – from Europe, North America, South America and Asia. The authors concluded that probiotics were better than placebo when measuring the number of participants experiencing episodes of acute URTI (at least one episode: odds ratio (OR) 0.53; 95% confidence interval (CI) 0.37 to 0.76, P value < 0.001),; the mean duration of an episode of acute URTI [mean difference (MD) −1.89; 95% CI −2.03 to −1.75, P value <0.001]; reduced antibiotic prescription rates for acute URTIs (OR 0.65; 95% CI 0.45 to 0.94) and cold-related school absence (OR 0.10; 95% CI 0.02 to 0.47).

As with all meta-analyses, there are limitations to the generalizability of pooled efficacy outcomes. However, even though the trials included in this analysis utilized heterogeneous interventions (different probiotic strains or combinations of strains), at variable dosages and durations, there at least appears to be a promising therapeutic benefit of taking probiotics for URTI prevention, as confirmed with strain-specific meta-analyses (Poon et al., 2020). Additionally, an economic modeling study applied to the US population, utilizing this same Cochrane data, concluded that probiotic use could achieve >54 million fewer days of infection, >2 million averted antibiotic courses, and >4 million avoided missed work days (Lenoir-Wijnkoop et al., 2019). The benefit of this in terms of cost savings, translated to the US population only, would represent approximately 1.4 billion USD annually. Continued research efforts to understand which probiotic strains, or combination of strains, achieve the most robust and reliable modulation of the immune system, and what the optimal dosing regimens and timing of intervention are, will enable the development of more effective probiotic therapies for viral URTIs and ultimately improve upon these figures further.

### Human Influenza Infection, Vaccine, and Probiotics

Human influenza viruses A, B, and C are RNA viruses of the family *Orthomyxoviridae* (Bouvier and Palese, 2008). These viruses primarily attach to, and replicate within, respiratory epithelial cells running from the upper (nasal cavity, oral cavity, pharynx, larynx) to lower respiratory tract (trachea, bronchi, bronchioles and alveoli) (Kalil and Thomas, 2019). Influenza usually causes only mild and uncomplicated disease, and most people recover without medical intervention. The predominant determinate of the severity of associated disease is the extent to which the lower respiratory tract becomes breached by the virus, risk factors for which include extremes of age (<5 years and > 65 years), Caucasians and comorbid chronic cardiorespiratory diagnoses (Kalil and Thomas, 2019). The disease occurs worldwide, and, in the northern hemisphere, annual flu epidemics (caused by influenza A and B viruses) occur during autumn and winter, affecting approximately 5%–15% of the population (Goeijenbier et al., 2017). Only influenza A viruses are known to cause flu pandemics. A recent study estimated that 291,243–645,832 seasonal influenza-associated respiratory deaths (4·0–8·8 per 100,000 individuals) occur each year globally, with the highest mortality rates among people aged 75 years or older (51·3–99·4 per 100,000 individuals) (Iuliano et al., 2018).

**Figure 2:**
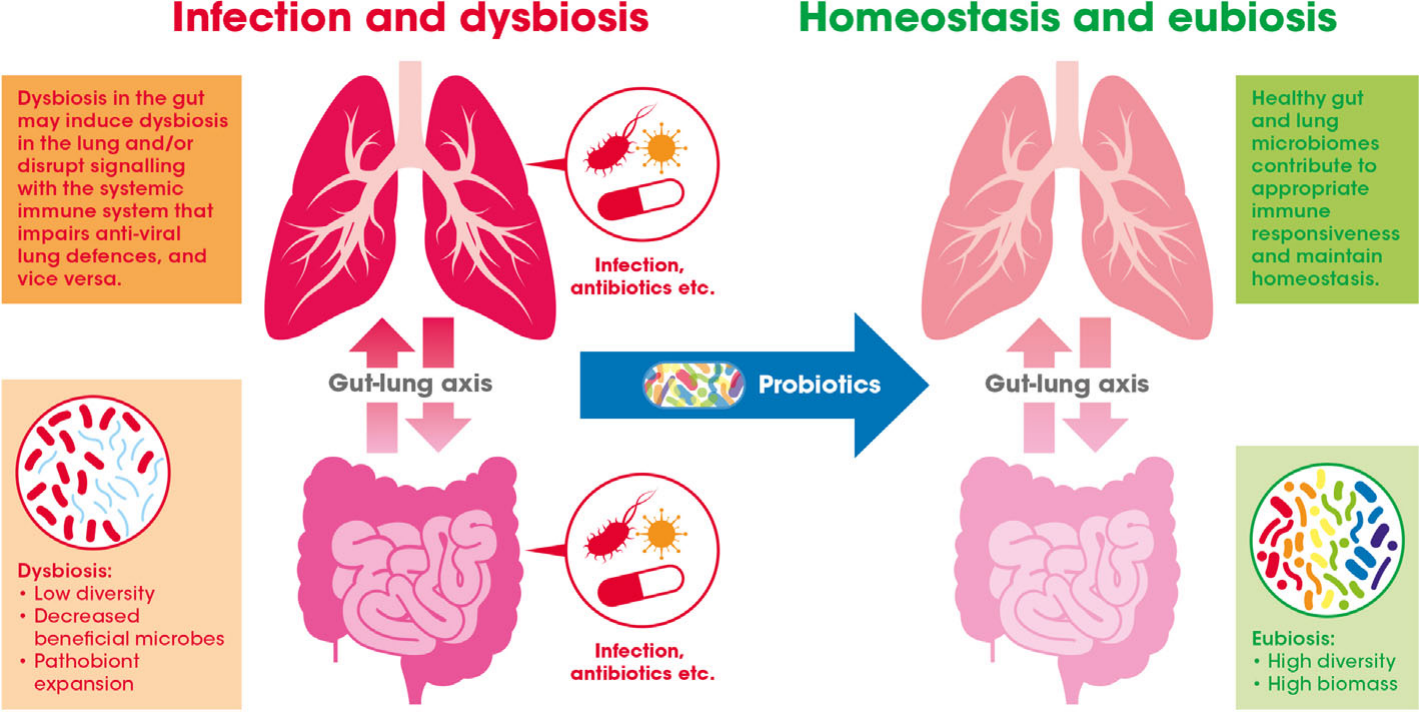
The gut-lung axis crosstalk in the context of viral respiratory infections: A healthy gut microbiome offers protection against respiratory infection by maintaining the normal immune response. This may be achieved through inter-organ signaling by numerous bacterially derived immunomodulating compounds [e.g., lipopolysaccharides (LPS), peptidoglycan, and short-chain fatty acids (SCFAs)]. Gastrointestinal (GI) dysbiosis caused by infection or antibiotic exposure, for example, may alter these microbiome generated signals (e.g., *via* the blood) and potentially impair antiviral immune responses in the lung. Introduction of beneficial bacteria (i.e., probiotics) may help to bolster or recover effective immune function in the GI tract and also indirectly in the lung.

Evidence suggests that the GI tract and respiratory system, along with their respective microbiomes, communicate with and influence each other – a relationship widely referred to as the gut-lung axis (see **Figure 2**) (Dumas et al., 2018).

Of particular interest is the observation that alterations in the GI microbiome appears to affect pulmonary immunity relevant to viral infections (Dumas et al., 2018). With regards to influenza infection, human data elucidating the exact nature of this phenomenon remains to be generated (Enaud et al., 2020). However, promising data from an influenza infection rodent model has revealed that the ingestion of probiotic strain *L. paracasei* CNCM I-1518 reduced susceptibility to influenza infection, reduced inflammatory cell infiltrates in the lungs and enhanced the speed of viral clearance (Belkacem et al., 2017). Furthermore, purified peptidoglycans (major cell wall components) from the strain were able to partially reproduce the effect of the intact cells. The mechanism by which these compounds might control viral infections was suggested by the authors to be related to recruitment of dendritic cells to the lungs. Additional rodent studies have shown a role for the microbiome in mediating IFN signatures in lung tissue, which can induce an environment refractory to early influenza virus replication thus blunting early influenza virus infection (Bradley et al., 2019). Moreover, both murine and human studies have revealed that antibiotics act to decrease pulmonary IgA production and increase the risk of pneumonia (Libertucci and Young, 2019). Indeed, animal studies have shown that antibiotic-eradication of the GI microbiome can impair both innate and adaptive immune responses and lead to impaired CD4+ and CD8+ T cell function (Chen et al., 2017). Furthermore, antibiotic induced dysbiosis in rodents reduces expression of toll like receptor 7 (TLR7) and NF- kB mRNA leading to impaired antiviral immunity, which was reversed with the administration of *Bifidobacterium* and *Lactobacillus* (*sensu lato*) probiotic strains (Wu et al., 2013). While we should be cautious before directly extrapolating from murine models to the human immune system, this research does demonstrate the intriguing interplay between the GI microbiome and the pulmonary immune system.

Public health measures, including encouraging those with respiratory symptoms to exercise social distancing and practice good hand hygiene practices, are vital to reduce the transmission of influenza, however vaccination remains the most effective measure to prevent infection. Vaccination acts to prime the adaptive immune system to recognize pathogens before infection, thus enabling a rapid response if and when subsequent exposure occurs. Vaccines can generally be divided into two broad categories: live attenuated vaccines (such as the rotavirus vaccine) and inactivated vaccines, of which there are a number of sub-groupings. Inactivated vaccines can be further sub-divided into: i) whole killed vaccines (e.g., polio); ii) toxoid vaccines, where vaccines are made with inactivated versions of toxins produced by the pathogen (e.g., diphtheria); iii) conjugate vaccines (e.g., meningitis c); and iv) recombinant vaccines (e.g., hepatitis B and HPV). Vaccines have substantially reduced the morbidity and mortality burden of communicable diseases. In developed countries the complete eradication of several infectious diseases, including smallpox are owed to vaccination. Moreover, it is estimated that 3 million lives are saved annually, thanks to vaccination (Toumi and Ricciardi, 2015). It is important to appreciate that numerous factors influence vaccine efficacy including nutrition, sex, age, genetics, and health status (Vlasova et al., 2019).

Unfortunately, vaccination frequently fails to provide adequate protection from infection; particularly in the elderly. In Europe only 44% of at risk individuals actually receive an annual influenza vaccination; far from the 75% target coverage rate set by the EU Council Recommendation in 2009 (Council of the European Union, 2009). Moreover, a recent review identified that vaccine effectiveness in adults aged >65 years was only 37% compared to 51% for working-age adults (Rondy et al., 2017). The reasons for this reduced effectiveness in the elderly is often attributed to immunosenescence. As detailed earlier, this process involves a decrease in a number of key immune cell types (e.g., T lymphocytes, natural killer cells, dendritic cells numbers, and loss of the diversity of B-cells). This results in a reduced capacity to mediate effective immune responses to vaccination and invading pathogens, which both increase susceptibility to infectious diseases (Pera et al., 2015).

One approach to improve influenza vaccines, and other vaccines, is to include adjuvants; substances that boost the immune response (Tregoning et al., 2018). Such substances are typically added to the vaccine such as aluminum salts. Along these lines there has been interest in utilizing probiotics (live microorganisms having beneficial effects on human hosts) to enhance response to vaccinations, based on substantial pre-clinical evidence identifying their immune-modulating properties (Rizzardini et al., 2012). In support of this, a recent systematic review of human clinical trials examining the influence of probiotics on vaccine response found evidence to suggest that certain probiotics increase the immune response to influenza vaccination, which raises the potential benefit for the elderly, in whom response to influenza vaccination (seroconversion) is impaired (Zimmermann and Curtis, 2017).

In a subsequent systematic review and meta-analysis of clinical trials using probiotics as adjuvants for influenza vaccination, hemagglutination inhibition (HI) antibody titers were examined (Yeh et al., 2018). The HI antibody titer test for influenza virus antibody in human sera has been shown to closely match those produced by virus neutralization assays and are predictive of protection. In this meta-analysis the authors found significantly higher HI titers in the probiotic group compared to control for both strain A/H1N1, A/H3N2 and strain B, with increases of 20%, 19.5%, and 13.6% respectively. This data suggests a promising role for microbiome manipulation to augment host antiviral immunity.

### Severe Acute Respiratory Syndrome Coronavirus 2 (SARS-CoV-2), the Microbiome and Probiotics

Angiotensin-converting enzyme 2 (ACE2) receptors, with which SARS-CoV-2 binds and invades human cells, is expressed not only in the airway but also the GI epithelium, and early reports indicated SARS-CoV-2 RNA was being detected in the stools of a significant fraction of patients (Wu et al., 2020). Productive infection has been demonstrated in enterocyte organoids *in vitro* (Lamers et al., 2020), and a recent meta-analysis of 17 studies confirmed that SARS-CoV-2 mRNA is detected in stools of almost half of coronavirus disease 2019 (COVID-19) patients; detection rate being markedly higher in severe cases and in those displaying GI symptoms (Gao et al., 2020). Diarrhea seems to be the most common GI symptom in children and adults, with a mean duration of 4.1 ± 2.5 days, while vomiting seems to be more prominent in children (Tian et al., 2020). Of note, viral mRNA detection in stools seems to be maintained in some patients after respiratory samples become negative (Wu et al., 2020).

These observations raise the possibility of the GI microbiome as a variable in the disease. In fact, an early limited case series of COVID-19 patients in China found reduced members of the genus *Lactobacillus* (*sensu lato*) and *Bifidobacterium* (Xu et al., 2020). Two subsequent studies comparing COVID-19 patients with both healthy controls and hospitalized patients with other respiratory infections, have found significant disturbances in the gut microbiota of COVID-19 patients (Zuo et al., 2020; Gu et al., 2020). More specifically, opportunistic pathogens like *Actinomyces*, *Erysipelaclostridium*, *Streptococcus*, *Veillonella*, *Rothia*, and *Enterobacter* were associated with COVID-19 diagnosis and/or severity among studies. Conversely, beneficial butyrogenic bacteria like *Faecalibacterium* and *Anaerostipes*, as well as of *Bifidobacterium* were inversely correlated with COVID-19 diagnosis and/or severity in both studies. Moreover, four specific bacteria (*Bacteroides dorei*, *Bacteroides thetaiotaomicron*, *Bacteroides massiliensis*, and *Bacteroides ovatus)*, known to downregulate ACE2 expression in the murine colon, were inversely correlated with SARS-CoV-2 mRNA load in feces (Zuo et al., 2020). The use of patients hospitalized for other conditions as controls in these studies provides methodological strength to these findings, and one of the studies also controlled for antibiotic co-therapy. However, a causative role for microbiota disturbances in COVID-19 severity remains to be confirmed.

Whatever the benefits offered by probiotics are, they are unlikely to have a direct effect on SARS-CoV-2 infection. In this regard, probiotics could facilitate a correction of the microbiota disturbances observed in some COVID-19 cases by inhibiting the growth of those specific opportunistic bacteria and/or facilitating the recovery of beneficial bacteria. Moreover, probiotics could boost immune system activity by direct cross-talk with immune cells and/or reduction of intestinal permeability, which has been proposed as one of the mechanisms behind the beneficial effect of probiotics on respiratory tract infections (Baud et al., 2020). Of note, SARS-CoV and SARS-CoV-2 cell entry results in downregulation of ACE2 (Verdecchia et al., 2020), and ACE2 deficiency has been shown to result in impaired tryptophan homeostasis (Hashimoto et al., 2012). Tryptophan metabolism across the serotonin, kynurenine and indole pathways, which are under control of the microbiota (Agus et al., 2018), has a strong influence on mucosal immunity and numerous systemic effects. Therefore, tryptophan-dependent pathways could provide a target for specific microbiota interventions in COVID-19. In this regard, administration of probiotic *L. plantarum* DR7 to healthy subjects has recently been shown to, compared to placebo, produce specific changes in the microbiota and altered serum tryptophan metabolic pathways (Liu G et al., 2020). However, it must be stressed that, at the time of writing, no probiotic strain has completed a randomized, placebo-controlled trial in COVID-19 patients, and therefore their use remains experimental.

### Microbiome, Viral Infections, and Probiotics in Critical Care

Critically ill patients are cared for in intensive care units (ICUs), otherwise known as critical care units (CCUs) or intensive therapy units (ITUs). Such patients have a high prevalence of at least one organ failure (51%–72%) at some point during their stay in ICU, with respiratory failure being the most prevalent (constituting 87% of patients with organ failure) (Pedersen et al., 2017). Organ failure is defined as organ dysfunction to such a degree that homeostasis cannot be maintained without external clinical intervention. Patients with respiratory failure, for instance, may be unable to breathe on their own and thus require mechanical ventilation.

Acute respiratory distress syndrome (ARDS) is a condition in which the lungs suffer severe, widespread injury, most commonly due to sepsis, leading to progressive respiratory failure. Pneumonia is the most common cause of sepsis (Dickson, 2017). ARDS was the deadliest complication of the pneumonia caused by severe acute respiratory syndrome (SARS) and H1N1 influenza viral epidemics (Hendrickson and Matthay, 2013). One of the most feared consequences of influenza infection is secondary bacterial infection (this is bacterial infection that occurs during or after an infection from another pathogen; commonly viruses) (Morris et al., 2017) and resulting ARDS, due to the substantial increase in morbidity and mortality. Respiratory failure may also be caused by exacerbation of chronic airway diseases (e.g., asthma, chronic obstructive pulmonary disease, and cystic fibrosis), and whereas bacteria play a controversial role in their pathogenesis, viruses are unambiguous precipitants (Dickson, 2017). Interestingly, oral consumption of a probiotic strain (*Lactocaseibacillus paracasei* CNCM I-1518) by mice infected with influenza A virus H3N2 was shown to relieve the burden of secondary bacterial infection from *Neisseria meningitidis* (Belkacem et al., 2018).

Recent studies have demonstrated substantial imbalances of the fecal microbiome in patients treated on the ICU (i.e., dysbiosis - an imbalance in either the species present on the functional capacity of those species in a particular ecological niche) (Wiersinga, 2017). This is an understandable consequence of both critical illness and the interventions of intensive care (e.g., antibiotics, proton pump inhibitors, dietary alterations, circadian rhythm disruption, ventilator support etc.). Beyond the impact on the body’s resident microbial populations, ICU interventions also increase intestinal permeability (e.g., non-steroidal anti-inflammatory drugs and parenteral feeding) and bacterial translocation – the movement of bacteria through the gut mucosa to normally sterile tissues (Dickson, 2017). This situation may exacerbate the immune dysregulation occurring in critical illness, thus increasing the burden of morbidity and mortality. Gut barrier dysfunction and dysbiosis in critical illness do however offer potential therapeutic targets. Numerous pre-clinical studies have shown that oral probiotics are capable of positively influencing gut barrier function and systemic immunity, and thus, in the case of pneumonia, may enhance the host’s ability to suppress and clear reproducing lung pathogens as they emerge (Bron et al., 2017) (Forsythe, 2014). Indeed, some promising evidence of therapeutic augmentation of the microbiome in critical illness exists (Schuijt et al., 2013). Recent reviews have concluded that the incidence of ventilator associated pneumonia (VAP - defined as an infectious inflammatory reaction of the lung that occurs after mechanical ventilation for >48 h) was safely reduced in patients taking probiotics (van Ruissen et al., 2019; Su M. et al., 2020). However, further large-scale studies are required given the limitations in the data currently available.

Even after survival of ARDS and sepsis, patients have an unfortunately high risk of re-admission in the months after discharge from ICU (Dickson, 2017). A recent publication examined admissions of nearly 11,000 patients (Americans, mean age 77 years) and reports a number of intriguing findings: 1) the rate of severe sepsis was 3.3 times greater in the 90 days after hospitalization (for all-cause hospitalization) than during all times before or after this period, and 2) readmission to hospital for severe sepsis was significantly higher (30%–70% more) in those patients who were admitted initially due to infection (Prescott et al., 2015). Dysbiosis, a well-established result of both infection and antibiotics, may partly explain this latter association. The authors hypothesize that periods of dysbiosis, during and after hospitalization, may impair the microbiome’s normal ability to modulate immunity and lead to increases in pathogenic organisms, which, when coupled with an overwhelming host inflammatory response, results in severe sepsis. The mechanisms underlying this so-called post intensive-care syndrome are poorly understood, but the contribution of a persistently altered microbiome needs to be explored (Dickson, 2017). It is well-established that even short courses of antibiotics can cause microbiome alterations that persist for weeks and months (Dethlefsen and Relman, 2011), and probiotics seem to offer some promising potential for re-establishing the microbiome following antimicrobial insult (Mcfarland, 2014). Further research is required to establish how quickly and completely the microbiome recovers following critical illness, and whether this process can be augmented with probiotics to realize clinically meaningful benefits.

## Conclusions

Advancements in the diagnosis, prevention, and treatment of infectious diseases in the 21^st^ century have brought profound social, economic and political benefits. However, respiratory tract infections, HIV and infectious gastroenteritis alone are predicted to result in an estimated 6.8 million premature deaths (0–69 years) in 2030 (Norheim et al., 2015). Therefore, infectious communicable diseases remain a significant threat to humankind requiring vigilance, surveillance and new interventions. Compared with antibiotics to treat bacterial infections, relatively few antiviral drugs have been developed to combat viral infections. This clearly represents a substantial unmet medical need, for which no single approach will suffice. In this instance, recent research suggests that the state of the human microbiome, in various niches, is relevant to viral pathogenesis. The intestinal microbiome, for example, appears to operate as a signaling hub that, through simultaneous integration of environmental, genetic and immune signals, is capable of affecting host metabolism, immunity and response to infection (Thaiss et al., 2016). Furthermore, recent advances in our understanding of the gut-lung and gut-liver (Milosevic et al., 2019) axes suggest novel means of protection against viral pathogens targeting extra-intestinal organs through manipulation of the GI microbiome (Dumas et al., 2018). The vaginal microbiome profile also appears to be highly relevant to viral pathogens, such as HIV and HPV, and thus represents a novel target to reduce the devastating burden of these diseases.

In the first part of this review we presented the evidence for a number of direct and indirect mechanisms by which bacterial strains might protect mammalian hosts from viral infections, such as modulating elements of both innate and adaptive immune systems. Continued progress in revealing and harnessing these mechanisms in humans will hopefully enable us to further reduce the transmission, morbidity and mortality of many notorious viral infections.

In the second part of this review we explored the research, with relevance to viral infections, demonstrating the sensitivity of the microbial component of the mammalian holobiont to disparate stressors including early life factors (e.g., caesarean section and restricted breast feeding) (Tamburini et al., 2016), antibiotic exposure (Dethlefsen and Relman, 2011), the aging process (e.g., immunosenescence and inflammaging) (Mangiola et al., 2018), unhealthy chronic dietary patterns and some inflammatory diseases (e.g., IBD, obesity and RA). The prospect of being able to bolster antiviral immunity through manipulation of the microbiome in these instances is enticing, however, causal relationships largely remain to be proven in human subjects.

In the third section we reviewed the current human clinical evidence for probiotics in the prevention and management of a range of viral pathogens relevant to the GI tract (rotavirus), liver (hepatitis B and C), cervicovaginal epithelium (HIV and HPV) and respiratory system (viral URTI pathogens, a particular focus on influenza, and SARS-CoV-2). Among these indications probiotics have been most extensively studied in the management of viral diarrhea (Szajewska et al., 2020) and URTIs (Hao et al., 2015) and, generally, the evidence supporting their efficacy and safety are positive. However, evidence of probiotic efficacy in the management of the viral infections detailed in this section requires further definitive research.

Human life is under constant threat from seasonal influenza epidemics and sporadic pandemics such as the one we are living through right now with COVID-19. Beyond social and hygiene measures it is clear that effective vaccines are required to provide definitive protection, however, past experience shows that the desired immune response to such interventions can be disappointing (Rondy et al., 2017). Evidence that probiotics may act as adjuvants to enhance the immune response to some vaccines is therefore promising, and should be explored in future large scale trials. Finally, we explored the impact that hospital intensive care has on the microbiome, considering that viral respiratory pathogens are responsible, either directly or indirectly, for a significant number of such admissions. Understanding the dynamics of this relationship opens novel treatment avenues with the potential to reduce the morbidity and mortality of these patients (van Ruissen et al., 2019; Su M. et al., 2020). Considering the excellent safety record and cost-effective nature of probiotics, the prospect of achieving even minimally clinically significant benefits makes further exploration worthwhile.

## Author Contributions

AH, RD, and VV designed and contributed to the writing of the manuscript. AO, JT, DO, JE, SB, and SD contributed to the writing of individual sections. AH was responsible for the whole manuscript and provided the final version of the review; VV and RD for proof reading final version. All authors contributed to the article and approved the submitted version.

## Funding

International Probiotics Association kindly supported the submission fee of this manuscript.

## Conflict of Interest

AH was previously employed by ADM Protexin Ltd., VV and RD are currently employed by ADM Protexin Ltd. AO is employed by DuPont Nutrition and Biosciences. DO is employed by Danone NutriciaResearch. JE is employed by AB-BIOTICS S.A. SB is employed by Lallemand Health Solutions, and SD is employed by Triphase Pharmaceuticals, Pvt Ltd.

The remaining author declares that the research was conducted in the absence of any commercial or financial relationships that could be construed as a potential conflict of interest.
